# NETs drive myocardial fibrosis in hypertension via an NF-κB/ferroptosis axis

**DOI:** 10.3389/fimmu.2025.1712786

**Published:** 2026-01-13

**Authors:** Qingxian Tu, Xiaowei Gong, Xiaoxi Yuan, Yiyue Tang, Runze Huang, Qianfeng Jiang, Wei Li

**Affiliations:** 1 Department of Cardiovascular Medicine, The Affiliated Hospital of Guizhou Medical University, Guiyang, Guizhou, China; 2 Department of Cardiovascular Medicine, The First People's Hospital of Zunyi/The Third Affiliated Hospital of Zunyi Medical University, Zunyi, Guizhou, China; 3 The Key Laboratory of Myocardial Remodeling Research, The Affiliated Hospital of Guizhou Medical University, Guiyang, Guizhou, China

**Keywords:** ferroptosis, hypertensive heart disease, myocardial fibrosis, neutrophil extracellular traps, NF-κB signaling

## Abstract

**Introduction:**

Hypertensive heart disease (HHD) is characterized by chronic pressure overload leading to myocardial remodeling and fibrosis. While inflammation and cell death pathways contribute to fibrogenesis, the mechanistic role of neutrophil extracellular traps (NETs) remains insufficiently understood. This study investigated whether NETs exacerbate myocardial fibrosis in spontaneously hypertensive rats (SHRs) through activation of the nuclear factor-κB (NF-κB) signaling pathway and ferroptosis.

**Methods:**

Male SHRs and normotensive Wistar-Kyoto (WKY) rats were used to assess blood pressure, cardiac function, and myocardial fibrosis via echocardiography, histology, and Western blotting. Transcriptomic profiling, immunofluorescence, and ELISA quantified NET-associated and ferroptosis-related markers. H9c2 cardiomyoblasts were treated with angiotensin II (Ang II) or isolated NETs, with or without DNase I, Ferrostatin-1 (Fer-1, ferroptosis inhibitor), or JSH-23 (NF-κB inhibitor). Gene and protein expression were analyzed by qPCR and Western blotting to elucidate NET-induced molecular mechanisms.

**Results:**

SHRs exhibited elevated systolic blood pressure, reduced ejection fraction, and marked myocardial fibrosis with increased collagen deposition. Transcriptomic and proteomic analyses revealed significant upregulation of NETs and ferroptosis-related genes, accompanied by NF-κB pathway enrichment. Myocardial and serum levels of citrullinated histone H3 and myeloperoxidase were elevated, confirming enhanced NET formation. In H9c2 cells, NETs induced ferroptotic features—elevated ACSL4, reduced FTH1 and GPX4—and activated NF-κB signaling, leading to increased expression of α-SMA, fibronectin, collagen II, and MMP3. DNase I pretreatment or inhibition of ferroptosis or NF-κB each mitigated these changes, with combined inhibition exerting the strongest suppressive effect.

**Discussion:**

These findings identify a pathogenic NETs/NF-κB/ferroptosis axis that drives hypertensive myocardial fibrosis. NETs promote oxidative stress and iron-dependent cell death in cardiomyocytes, amplifying inflammatory and profibrotic signaling. Therapeutic targeting of NETs formation or downstream ferroptotic and NF-κB pathways may thus attenuate fibrotic remodeling and preserve cardiac function in HHD. This study provides mechanistic insight into how sterile inflammation orchestrates myocardial injury and highlights novel intervention strategies against hypertensive cardiac fibrosis.

## Graphical Abstract

**Figure:**
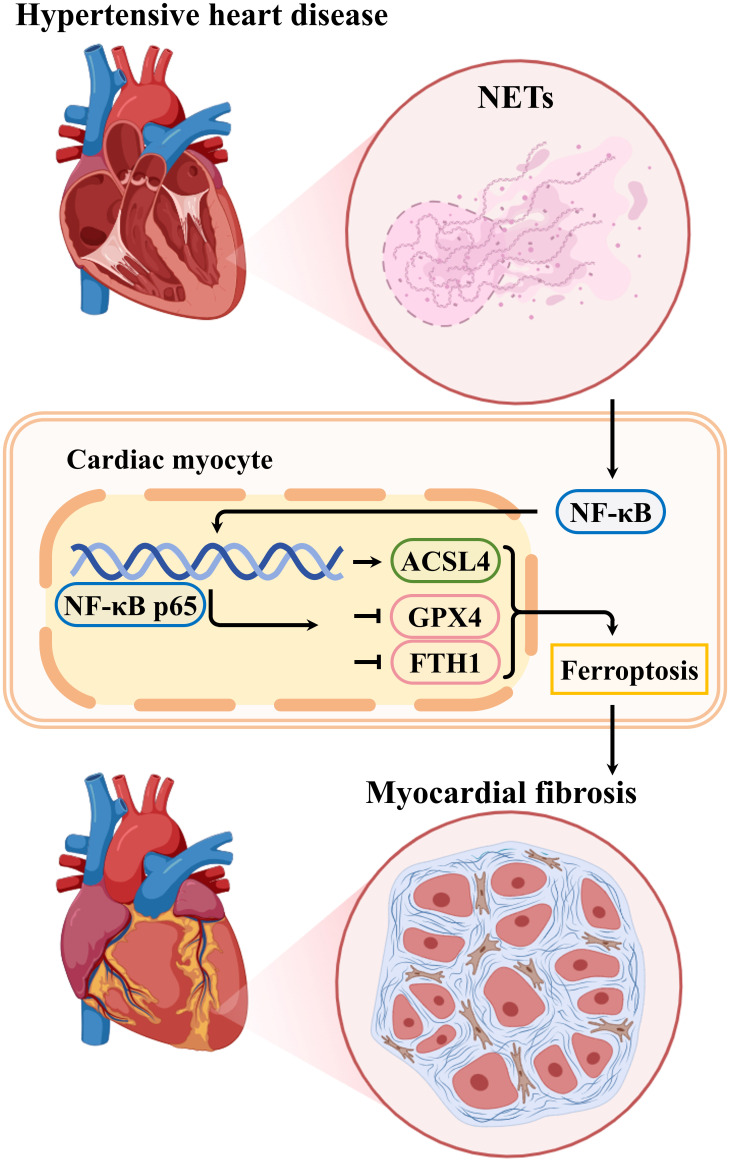

## Introduction

Hypertensive heart disease (HHD) remains a major global health burden and a leading cause of morbidity and mortality associated with chronic hypertension (1, 2). Persistent elevation of arterial pressure triggers a cascade of adaptive and maladaptive myocardial responses, culminating in cardiac remodeling characterized by left ventricular hypertrophy and microvascular dysfunction (3–5). Among these changes, myocardial fibrosis—characterized by excessive deposition of extracellular matrix and interstitial collagen—is a hallmark pathological feature that contributes to diastolic dysfunction and progression to heart failure with preserved ejection fraction (HFpEF) (6, 7). Although various neurohumoral and mechanical factors are implicated in fibrosis development, recent studies have highlighted the critical contribution of sterile inflammation and regulated cell death pathways in driving fibrogenic processes (8–10).

Ferroptosis, an iron-dependent form of regulated cell death driven by lipid peroxidation, has emerged as a pivotal contributor to cardiac injury and fibrotic remodeling in diverse cardiovascular settings (11–13). Accumulating evidence suggests that ferroptosis not only results in cardiomyocyte loss but also stimulates the release of damage-associated molecular patterns (DAMPs), thereby promoting inflammatory responses and fibroblast activation (14–16). The involvement of ferroptosis in hypertension-related myocardial fibrosis remains an area of active investigation, particularly regarding its initiating triggers and downstream signaling cascades (17, 18).

Neutrophil extracellular traps (NETs)—web-like structures composed of DNA, histones, and granule proteins—are released by activated neutrophils and have been implicated in both innate immunity and tissue injury (19, 20). Beyond their antimicrobial function, NETs have been shown to contribute to cardiovascular pathology by inducing endothelial dysfunction, exacerbating oxidative stress, and facilitating fibroblast activation (21–23). In hypertensive models, NET formation correlates with increased myocardial damage and fibrotic remodeling, although the molecular mediators linking NETs to fibrosis remain incompletely elucidated.

One such mediator is the nuclear factor kappa-light-chain-enhancer of activated B cells (NF-κB), a transcription factor that orchestrates proinflammatory and profibrotic gene expression (24, 25). Activation of NF-κB in cardiac cells leads to upregulation of cytokines, chemokines, and matrix metalloproteinases, thereby establishing a microenvironment conducive to fibrosis (26, 27). Notably, both NETs and ferroptosis have been shown to activate the NF-κB signaling pathway, suggesting a mechanistic convergence whereby neutrophil-derived products and iron-catalyzed lipid damage jointly promote fibrogenesis via NF-κB-mediated transcriptional reprogramming (28–30). Despite these advances, a direct mechanistic link between NETs, NF-κB activation, ferroptosis, and myocardial fibrosis in hypertension remains unexplored.

This study investigates the hypothesis that NETs exacerbate myocardial fibrosis in spontaneously hypertensive rats by activating NF-κB signaling, thereby promoting cardiomyocyte ferroptosis and fibroblast transdifferentiation. We provide novel evidence that NET degradation or pathway inhibition attenuates fibrosis through suppression of NF-κB-dependent ferroptotic responses, highlighting the NETs/NF-κB/ferroptosis axis as a potential therapeutic target in HHD.

## Materials and methods

### Cell culture establishment from cryopreservation

H9c2 rat cardiomyoblast cells (Catalog #LJS-r012, Lingjiesi Biotech, Wuhan, China) were routinely cultured in Dulbecco’s Modified Eagle Medium (DMEM) supplemented with 1.5 g/L sodium bicarbonate, 10% (v/v) premium-grade fetal bovine serum, and 1% (v/v) penicillin-streptomycin antibiotic solution. Cells were maintained in a humidified incubator at 37 °C under a controlled atmosphere of 5% CO_2_ and 95% air, with relative humidity maintained between 70% and 80%. For recovery from cryopreservation, vials containing 1 mL frozen cell suspension were rapidly thawed by gentle agitation in a 37 °C water bath. The thawed suspension was immediately transferred into a sterile centrifuge tube containing 4–6 mL of pre-warmed complete culture medium and mixed thoroughly. The cell suspension was then centrifuged at 1000 revolutions per minute (RPM) for 3–5 minutes to pellet the cells. Following centrifugation, the supernatant was carefully aspirated and discarded without disturbing the cell pellet. The cell pellet was then resuspended in an adequate volume of fresh complete medium to achieve a homogeneous cell suspension. This cell suspension was transferred to a culture flask containing an additional 6–8 mL of complete medium. The flask was placed in the 37 °C incubator and allowed to incubate undisturbed overnight to facilitate cell adherence and initial recovery. Cellular morphology, attachment efficiency, and approximate confluence were assessed using phase-contrast microscopy approximately 24 hours post-thaw to confirm viability and readiness for subsequent experimental procedures or further passaging.

### Animal subjects and dietary treatment

A total of 12 male SPF-grade rats, consisting of 6 spontaneously hypertensive rats and 6 Wistar rats, all aged 6–8 weeks, were procured from Vital River Laboratory Animal Technology. The spontaneously hypertensive rats were randomly allocated into two experimental groups (n=6 per group): a control group maintained on a standard diet containing 0.3% NaCl for 8 weeks without any intervention, and a model group subjected to a high-salt diet with 8% NaCl for 8 weeks to induce hypertensive pathology (31, 32). Age-matched Wistar-Kyoto (WKY) rats, the normotensive control strain for SHRs, were included under the same conditions and utilized as controls in subsequent experiments. All animals were housed and handled according to institutional guidelines and with prior approval from the Laboratory Animal Welfare & Ethics Committee of Zunyi Medical University (Approval No. ZMU21-2403-311) throughout the 8-week period.

### Cardiac ultrasound assessment with physiological monitoring

Following a 12-hour fasting period with ad libitum water access, rats were anesthetized using isoflurane delivered in oxygen (induction: 3% at 1 L/min; maintenance: 1.5–2% at 500–700 mL/min). Animals were positioned supine on a heating pad, and core body temperature was continuously monitored via a rectal probe and maintained at 37.0 ± 0.5 °C. Limb electrodes with conductive gel were affixed for electrocardiogram (ECG) recording. The left parasternal chest region was depilated using a chemical depilatory agent and cleaned with water to ensure adequate acoustic coupling.

Cardiac ultrasound imaging was performed using the Vevo 2100 high-resolution system (FUJIFILM VisualSonics) equipped with an MS-250 transducer (central frequency: 21 MHz). All examinations were conducted in a temperature-controlled environment (22–25 °C, humidity 40–60%). Two-dimensional (B-mode) imaging was initiated with the following parameters: imaging depth set to 15–20 mm, gain adjusted to optimize myocardial border delineation, and frame rate maintained at ≥300 frames per second to accommodate the high heart rate of rodents. Standard parasternal long-axis (PLAX) and short-axis (PSAX) views were acquired. For the PLAX view, the transducer was positioned at the left sternal border (approximately the 3rd–4th intercostal space) with the beam oriented parallel to the long axis of the left ventricle, ensuring clear visualization of the aortic root, mitral valve, and left atrium. The PSAX view was obtained by rotating the transducer 90°clockwise at the papillary muscle level, where the left ventricle appears circular and both papillary muscles are distinctly visible.

M-mode recordings were subsequently obtained from the PSAX view at the papillary muscle level, with the M-mode cursor aligned perpendicular to the interventricular septum and left ventricular posterior wall. Left ventricular internal diameter at end-diastole (LVIDd) and end-systole (LVIDs), interventricular septal thickness (IVS), and left ventricular posterior wall thickness (LVPW) were measured from three consecutive cardiac cycles. Left ventricular ejection fraction (LVEF) and fractional shortening (FS) were calculated using the built-in Vevo LAB software according to the Teichholz formula. All measurements were performed by an experienced operator blinded to the group allocation.

### Reagents and materials

All chemical reagents, inhibitors, antibodies, transfection reagents, and assay kits utilized in this study were sourced from commercial suppliers with specific catalog numbers as follows: Angiotensin II (Ang II, HY-13948), Ferrostatin-1 (HY-100579), and JSH-23 (HY-13982) were procured from MedChemExpress (MCE); Phorbol 12-myristate 13-acetate (PMA, P1585) was obtained from Sigma-Aldrich; DNase I (HY-108882) was acquired from MCE; Lipofectamine 2000 transfection reagent (11668-019) was purchased from Invitrogen. Primary antibodies included those against GPX4 (LJS-D-6701), FTH1 (LJS-D-6278), and ACSL4 (LJS-D-12141), sourced from Lingjiesi Biotech (Wuhan, China); α-Smooth Muscle Actin (α-SMA, 19245T) from Cell Signaling Technology; Collagen Type II (Ab34172) from Abcam; Fibronectin (15613-1-AP), Matrix Metalloproteinase 3 (MMP3, 66338-1-Ig), Phospho-NF-κB p65 (82335-1-RR), NF-κB p65 (10745-1-AP), Histone H3 (17168-1-AP), and Myeloperoxidase (MPO, 66177-1-Ig) from Proteintech (China); and GAPDH (GB12002) from ServiceBio. Oxidative stress and antioxidant markers were quantified using commercially available assay kits for Malondialdehyde (MDA, A003-1-2), Superoxide Dismutase (SOD, A001-3-2), and Reduced Glutathione (GSH, A006-2-1), all purchased from Nanjing Jiancheng Bioengineering Institute.

### Ang II stimulation of H9c2 cells and induction of pro-fibrotic signaling

H9c2 cells were seeded in 96-well plates at a density of 1 × 10^4^ cells per well and cultured until reaching 70% confluency. The cells were then incubated with 10^-6^ M Ang II for 24 hours to simulate hypertensive stress and induce a profibrotic signaling response, as previously described in studies investigating cardiomyocyte contribution to fibrosis (33–35), after which they were harvested for subsequent analysis.

### Experimental group design for H9c2 cell treatments

H9c2 cells were systematically allocated into seven experimental groups to investigate specific molecular pathways. The control group remained untreated throughout the experiment. The model group was exposed to 10⁻^6^ M Ang II to establish a disease phenotype. The NETs group received 500 μL of neutrophil extracellular traps (NETs) at 180 ng/mL for 24 hours (36). For mechanistic interrogation, the NETs+DNase I group was treated with 200 μL of NETs (180 ng/mL) pre-digested with 20 μg/mL DNase I. The NETs+ferroptosis inhibitor group received identical NETs treatment co-administered with 5 μM ferrostatin-1 (Fer-1) (17). Similarly, the NETs+NF-κB inhibitor group was treated with NETs plus 30 μM JSH-23 (37). The combinatorial intervention group (NETs+ferroptosis inhibitor+NF-κB inhibitor) was exposed to NETs concurrently with both 5 μM Fer-1 and 30 μM JSH-23. All treatments were maintained for 24 hours under standardized culture conditions prior to downstream analysis.

### Isolation and quantification of neutrophil extracellular traps

Neutrophils were isolated from fresh rat peripheral blood anticoagulated with EDTA using a commercial separation kit (P9200, Solarbio, Beijing, China). Briefly, 1 ml of anticoagulated blood was mixed 1:1 with the provided whole blood and tissue diluent and carefully layered onto Solution A. Centrifugation was performed at 500 × g (approximately 1800 rpm, radius 15 cm) for 25 minutes using a horizontal rotor. Following centrifugation, the layer containing enriched neutrophils (the slightly turbid third layer above the red blood cell pellet) along with the red blood cell layer (fourth layer) was collected, discarding the top plasma and mononuclear cell layers. This collected fraction was washed once with cell wash solution (10 ml), centrifuged at 500 × g for 30 minutes, and the pellet was treated with red blood cell lysis buffer. After three subsequent washes to remove erythrocyte debris, the final neutrophil pellet was obtained. For NET induction, isolated neutrophils were resuspended at 4×10^6^ cells/ml and stimulated with 100 nM phorbol 12-myristate 13-acetate (PMA) for 3 hours at 37 °C. The culture medium was then collected and centrifuged at 450 × g for 10 minutes to remove cellular components; the resulting supernatant, containing released NETs, was subjected to further centrifugation at 18,000 × g for 10 minutes at 4 °C. The pelleted NETs were resuspended in phosphate-buffered saline (PBS). NET-derived DNA content was quantified using a Nano-100 spectrophotometer. NET formation was confirmed by immunofluorescence staining for established NET markers citrullinated histone 3 (Cit-H3) and myeloperoxidase (MPO).

### RNA isolation and real-time quantitative PCR analysis

Total RNA was extracted from harvested cell pellets using the Ultrapure RNA Kit (CW0597S, CWBIO, Taizhou, Jiangsu, China). Cell pellets were homogenized in 1 mL TRIzon Reagent using a tissue homogenizer, followed by vortexing and incubation at room temperature for 5 min to ensure complete lysis and dissociation of nucleoprotein complexes. Subsequently, 200 μL chloroform was added, and samples were vigorously vortexed for 15 sec and incubated at room temperature for 2 min. Phase separation was achieved by centrifugation at 12,000 rpm for 10 min at 4 °C. The upper aqueous phase, containing RNA, was carefully transferred to a new RNase-free tube and mixed with an equal volume of 70% ethanol (prepared with RNase-free water). This mixture was loaded onto the provided Spin Columns RM and centrifuged at 12,000 rpm for 30 sec; the flow-through was discarded. The column was washed with 350 μL Buffer RW1 (centrifugation at 12,000 rpm for 30 sec). To eliminate genomic DNA contamination, 80 μL of a freshly prepared DNase I mixture (containing 52 μL RNase-free water, 8 μL 10× Reaction Buffer, and 20 μL DNase I) was added directly to the column membrane and incubated at 20–30 °C for 15 min. After incubation, the column was washed again with 350 μL Buffer RW1 (12,000 rpm, 1 min), followed by two sequential washes with 500 μL Buffer RW1 (12,000 rpm, 20 sec each). Residual ethanol was removed by centrifuging the column at 12,000 rpm for 2 min and air-drying at room temperature. Purified RNA was eluted by adding 30–50 μL RNase-free water to the column membrane, incubating for 1 min at room temperature, and centrifuging at 12,000 rpm for 1 min. Eluted RNA was stored at −80 °C to prevent degradation. Complementary DNA (cDNA) was synthesized from this RNA using the TransGen Biotech (Beijing, China) cDNA synthesis kit. For qPCR, reactions were assembled using Sangon Biotech (Shanghai, China)-designed primers (sequences in **Table 1**), SybrGreen qPCR Master Mix, cDNA template, and nuclease-free water. Gene expression analysis employed the 2^−ΔΔ^*^Ct^* method using internal reference genes for normalization.

**Table 1:** Primer sequences.

Primer	Sequence (5’-3’)
α-SMA-F	ACCATCGGGAATGAACGCTT
α-SMA-R	CTGTCAGCAATGCCTGGGTA
Fibronectin-F	GTAACCGTCAGGCACCTCAA
Fibronectin-R	TCCTCACATCCTTCTTCTGCTG
Collagen Type II-F	ACACTGGGAATGTCCTCTGC
Collagen Type II-R	ACTCTCCGAAGGGGATCTCG
MMP3-F	ATGGGCCTGGAATGGTCTTG
MMP3-R	TGTGGGAGGTCCATAGAGGG
GAPDH-F	GGAAAGCTGTGGCGTGAT
GAPDH-R	TCCACAACGGATACATTGGG

### EdU staining for cell proliferation assessment

Cells were seeded at a density of 1 × 10^5^ cells per well in 24-well plates and subjected to experimental treatments as per the study protocol. Cell proliferation was evaluated using the BeyoClick™ EdU Cell Proliferation Kit (Catalogue No. C0071S, Beyotime, Shanghai, China). A 2× EdU working solution (20 μM) was prepared by diluting the stock EdU (10 mM) 1:500 in cell culture medium, pre-warmed to 37 °C, and added in equal volume to achieve a final concentration of 10 μM. After a 2-hour incubation at 37 °C, the medium was removed, and cells were fixed with 0.5 ml of 4% paraformaldehyde at room temperature for 15 minutes. Following fixation, cells were washed three times with 0.5 ml phosphate-buffered saline (PBS) for 3–5 minutes per wash. Permeabilization was performed using 0.5 ml of PBS containing 0.3% Triton X-100 for 10–15 minutes at room temperature, after which cells were washed once or twice with PBS. The Click reaction solution was prepared and applied according to the manufacturer’s instructions to detect EdU incorporation. For nuclear counterstaining, cells were incubated with 300 μl of DAPI solution in the dark at room temperature for 10 minutes, followed by three PBS washes. Imaging was conducted using fluorescence microscopy to quantify proliferating cells.

### Protein extraction and Western blotting analysis

Protein lysates were prepared from collected cell or tissue homogenates by resuspension in 500 μL of RIPA buffer supplemented with 1 mM PMSF, followed by incubation on ice for 30 minutes. The lysates were then centrifuged at 12,000 rpm for 10 minutes at 4 °C to pellet insoluble debris, and the resulting supernatant containing soluble proteins was collected. Total protein concentration within these supernatants was determined using a BCA protein assay kit according to the manufacturer’s instructions. Sample protein concentrations were subsequently adjusted to an optimal range and denatured by mixing with 5× Laemmli loading buffer, boiling for 5–10 minutes, and incubating on ice for 5–10 minutes before storage at -20 °C until further analysis. Proteins were resolved by SDS-PAGE using resolving gels with acrylamide concentrations between 10% and 15%, selected based on the molecular weight of the target proteins. Denatured protein samples (20-40 μg total protein per lane) were electrophoresed initially at 80 V for 30 minutes, followed by 120 V for 1–2 hours. Separated proteins were then transferred onto PVDF membranes using a wet transfer system at 300 mA for 60 minutes under constant cooling with an ice bath; PVDF membranes were pre-activated by brief immersion in methanol for 30 seconds prior to transfer. After transfer, membranes were briefly rinsed once with TBST and subsequently blocked by incubation in blocking buffer with gentle agitation at room temperature for 2 hours, followed by another TBST wash. Primary antibody incubations were performed overnight at 4 °C using appropriately diluted antibodies specific for the target proteins (typically 1:1000) and GAPDH (1:2000) diluted in PBS, antibody diluent, or blocking buffer. Membranes were washed three times for 10 minutes each with TBST. This was followed by incubation with horseradish peroxidase (HRP)-conjugated secondary antibodies (either goat anti-mouse IgG or goat anti-rabbit IgG, both at 1:2000 dilution) for 1 hour at room temperature with gentle shaking. After five 10-minute TBST washes, protein bands were visualized by applying enhanced chemiluminescence (ECL) substrate to the membranes and capturing the chemiluminescent signal via an imaging system.

### Immunofluorescence staining

Tissue specimens underwent sequential dehydration in a graded ethanol series (75% to absolute ethanol) using an automated tissue processor, followed by clearing in xylene and infiltration with paraffin wax at 65 °C. Paraffin-embedded tissues were sectioned at 4 μm thickness using a Leica microtome, mounted on adhesive slides, and baked at 60 °C for 3 hours. Deparaffinization was performed through xylene and a reverse ethanol gradient, then rehydrated in distilled water. Antigen retrieval utilized heat-induced epitope retrieval in 0.01 M EDTA buffer (pH 9.0) via an electric ceramic hob: samples were boiled for 15 minutes and cooled to room temperature before PBS washes. Non-specific binding was blocked with normal goat serum (30 minutes, room temperature). Sections were incubated overnight at 4 °C with primary antibodies: rabbit anti-histone H3 antibody, followed by Cy3-conjugated goat anti-rabbit IgG (37 °C, 1 hour). After repeated blocking, mouse anti-myeloperoxidase (MPO) antibody was applied overnight at 4 °C, then detected with FITC-conjugated goat anti-mouse IgG (37 °C, 1 hour). All post-secondary antibody steps were conducted under minimal light. Nuclei were counterstained with DAPI (5 minutes), and slides were mounted with antifade medium for fluorescence microscopy analysis.

### RNA sequencing

Total RNA was extracted from myocardial tissue samples. Eukaryotic mRNA was enriched using magnetic beads conjugated with Oligo(dT). The purified mRNA was fragmented by incubation in fragmentation buffer. Using these fragmented mRNAs as templates, first-strand cDNA synthesis was performed with random hexamer primers. Second-strand cDNA synthesis was subsequently carried out by adding buffer, dNTPs, RNase H, and DNA polymerase I. The resulting double-stranded cDNA underwent end repair, poly(A) tailing, and ligation of Illumina-compatible sequencing adapters. Size selection and purification of the adapter-ligated products were achieved using magnetic beads. Finally, PCR amplification was performed to generate the final sequencing libraries. All steps were performed by Wuhan Lingsi Biotechnology Co., Ltd.

### Statistical analysis

All experiments were performed with at least three independent replicates to ensure the reliability and reproducibility of the findings. Data are presented as mean ± standard deviation (SD). Prior to statistical testing, the normality of all data distributions was assessed using the Shapiro-Wilk test, and the homogeneity of variances was verified using Levene’s test. For comparisons between two groups that met both normality and variance homogeneity assumptions, an unpaired, two-tailed Student’s *t*-test was employed. For comparisons among three or more groups, one-way analysis of variance (ANOVA) was applied, followed by Tukey’s honestly significant difference (HSD) *post-hoc* test for multiple comparisons.

In instances where data violated the normality assumption and/or the assumption of equal variances, appropriate non-parametric tests were used. Specifically, the Mann-Whitney U test was used for two-group comparisons, and the Kruskal-Wallis test was used for multiple groups, followed by Dunn’s *post-hoc* test for pairwise comparisons. All statistical analyses were conducted using GraphPad Prism software (version 9.5.0, GraphPad Software, USA). A *p*-value of less than 0.05 was considered statistically significant.

## Results

### Cardiovascular dysfunction and myocardial remodeling in spontaneously hypertensive rats

**Figure 1:**
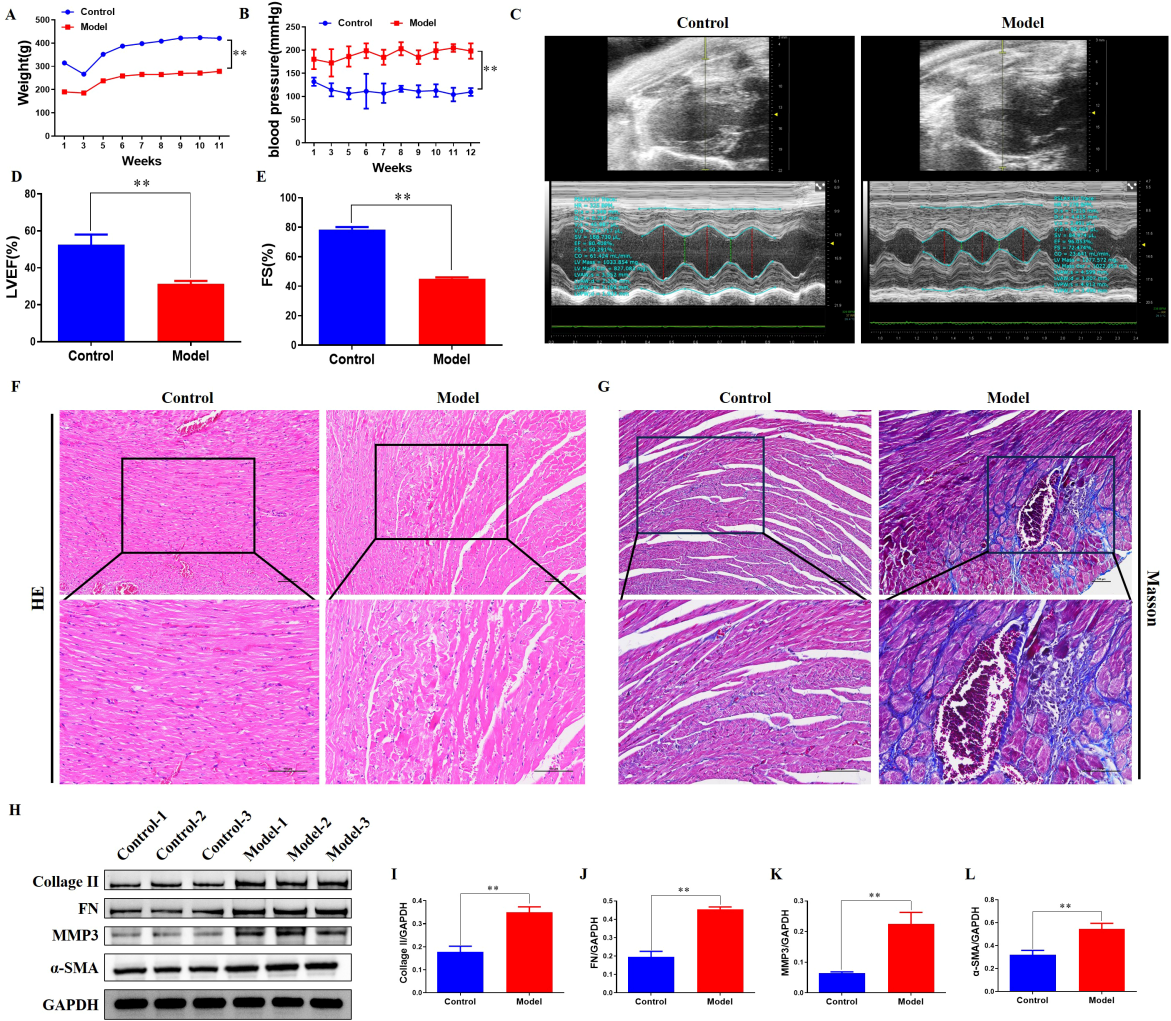
Cardiovascular dysfunction and pathological myocardial remodeling in spontaneously hypertensive rats. **(A)** Body weight changes in control and spontaneously hypertensive rats (**P < 0.01). **(B)** Serial systolic blood pressure measurements in control and spontaneously hypertensive rats (**P < 0.01). **(C)** Representative echocardiographic images. **(D)** Quantification of left ventricular ejection fraction (LVEF) (**P < 0.01). **(E)** Measurement of left ventricular fractional shortening (FS) demonstrating significant impairment in spontaneously hypertensive rats versus controls (**P < 0.01). **(F)** Representative hematoxylin and eosin-stained myocardial sections. **(G)** Masson’s trichrome-stained myocardial sections. **(H)** Representative immunoblots of fibrosis-related proteins. **(I)** Collagen II expression quantification (**P < 0.01). **(J)** Fibronectin expression quantification (**P < 0.01). **(K)** MMP3 expression quantification (**P < 0.01). **(L)** a-SMA expression quantification (**P < 0.01).

Spontaneously hypertensive rats exhibited significant physiological alterations compared to normotensive control animals at the same age. The body weight of hypertensive animals was significantly lower than that of the normotensive controls (**Figure 1A**). Concurrently, systolic blood pressure was markedly elevated in the hypertensive animals compared to controls (**Figure 1B**). Assessment of cardiac function by echocardiography revealed pronounced impairment in the hypertensive group. Representative echocardiographic images illustrating these findings are shown in **Figure 1C**. Left ventricular ejection fraction (LVEF) was significantly reduced in hypertensive animals compared to controls (**Figure 1D**). Similarly, left ventricular fractional shortening (FS) was significantly lower in the hypertensive group than in the normotensive group (**Figure 1E**).

Histopathological examination of myocardial tissue demonstrated substantial structural derangement in the hypertensive animals. Hematoxylin and eosin (HE) staining showed well-organized cardiomyocytes with eosinophilic (pink) cytoplasm, central oval nuclei, and sparse interstitium in control myocardium. In contrast, model myocardium displayed extensive fibrosis, characterized by widened interstitium with abundant homogeneous pink collagen deposits. Cardiomyocytes exhibited fragmentation and reduction, accompanied by nuclear pyknosis in the remaining cells (**Figure 1F**).

Masson’s trichrome staining revealed significant differences in myocardial collagen deposition between the control and hypertensive animals. In myocardial tissue sections from control rats, delicate blue-stained collagen fibers were sparsely distributed within the interstitium, and the continuous red-stained myocardium predominated. Collagen volume fraction (CVF) quantification confirmed this minimal fibrosis, averaging approximately 3% (**Figure 1G**). In stark contrast, myocardial sections from hypertensive animals exhibited profound alterations characteristic of extensive fibrosis. Thick layers of blue-stained collagen formed prominent perivascular cuffs around blood vessels. Furthermore, a diffuse proliferation of blue-stained reticular fibers permeated the myocardial interstitium. This pervasive collagen deposition disrupted the myocardial architecture, isolating cardiomyocytes into island-like structures. Quantitative assessment corroborated the severe histopathological changes, demonstrating a substantial increase in CVF to approximately 22% in the hypertensive animals (**Figure 1G**).

Complementing the histopathological evidence, Western blot analysis of key myocardial fibrosis markers consistently demonstrated elevated expression levels in the hypertensive animals compared to controls. Protein expression of Collagen type II (Collagen II), Fibronectin (FN), Matrix Metalloproteinase-3 (MMP3), and a-smooth muscle actin (a-SMA) were all significantly higher in myocardial tissue lysates prepared from hypertensive animals (**Figures 1H–L**). These molecular findings confirm the activation of fibrotic pathways at the protein level in the hypertensive myocardium.

### Transcriptomic profiling and experimental validation reveal enhanced NET formation and fibrotic marker expression in hypertensive myocardium

**Figure 2:**
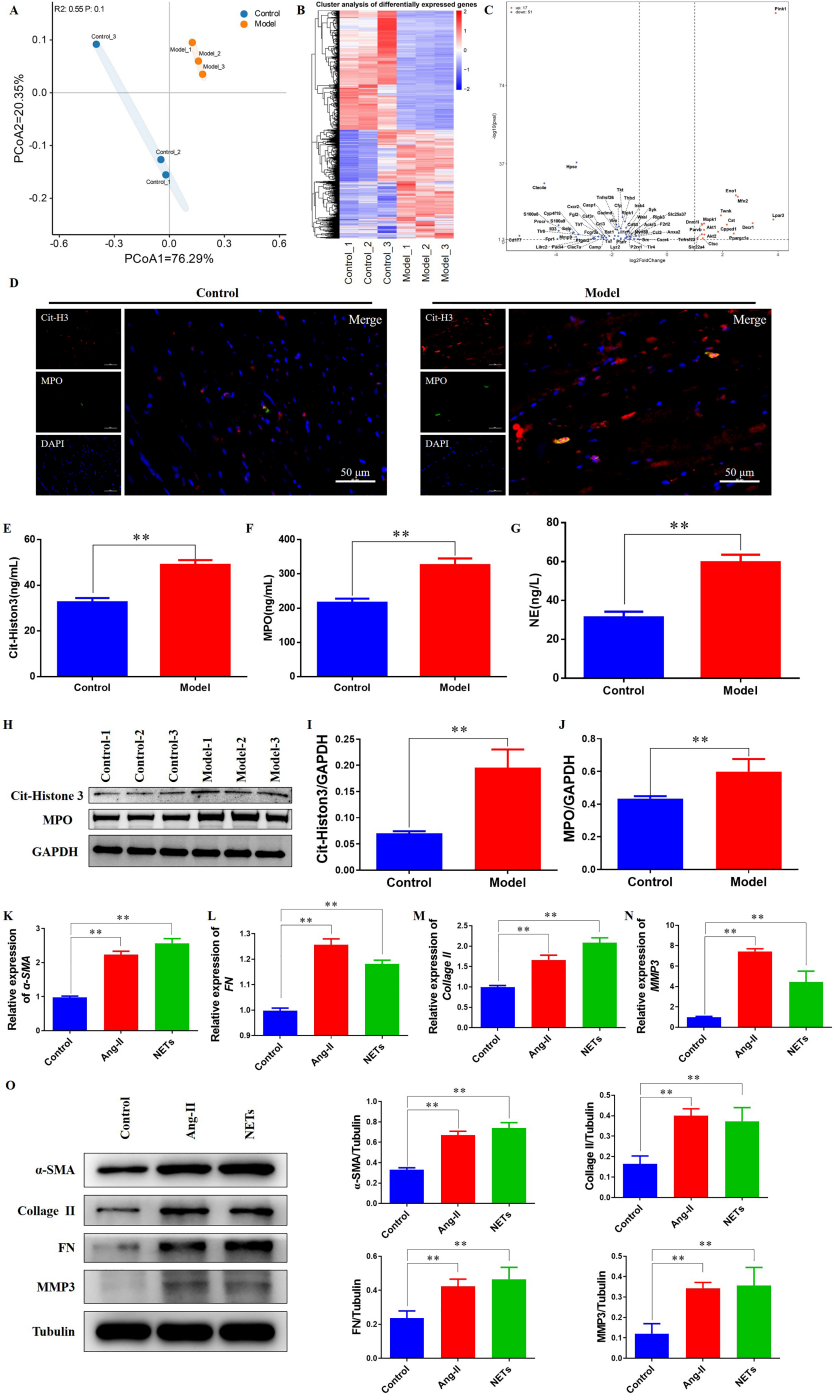
Enhanced NET formation and fibrotic marker expression in hypertensive myocardium. **(A)** Principal component analysis (PCA) showing separation between Control and hypertensive animals. **(B)** Heatmap of NETs-related genes dysregulated in hypertensive animals. **(C)** Volcano plot of differentially expressed genes. **(D)** Immunofluorescence staining of myocardial tissue showing increased Cit-H3 and MPO expression in hypertensive animals. **(E)** Serum Cit-H3 concentrations elevated in hypertensive animals by ELISA (**P < 0.01). **(F)** Serum MPO concentrations elevated in hypertensive animals by ELISA (**P < 0.01). **(G)** Serum NE concentrations elevated in hypertensive animals by ELISA (**P < 0.01). **(H)** Representative western blots of Cit-H3 and MPO in myocardial tissue. **(I)** Quantitative analysis of Cit-H3 protein expression in myocardial tissue (**P < 0.01). **(J)** Quantitative analysis of MPO protein expression in myocardial tissue (**P < 0.01). **(K)** mRNA expression levels of α-SMA (**P < 0.01). **(L)** mRNA expression levels of FN (**P < 0.01). **(M)** mRNA expression levels of Collagen II (**P < 0.01). **(N)** mRNA expression levels of MMP3 (**P < 0.01). **(O)** Protein expression levels of α-SMA, FN, Collagen II, and MMP3 determined by Western blotting (**P < 0.01).

Principal component analysis of transcriptomic data demonstrated a clear separation between the Control and spontaneously hypertensive (Model) groups, indicating distinct global gene expression patterns (**Figure 2A**). Heatmap analysis revealed 3,411 genes significantly upregulated and 3,718 genes significantly downregulated in the hypertensive animals compared with the Control group (**Figure 2B**). Notably, among neutrophil extracellular trap (NET)-associated genes, 17 were upregulated and 51 were downregulated in the hypertensive animals, as highlighted in the volcano plot (**Figure 2C**).

Immunofluorescence staining showed markedly increased expression of NET markers, citrullinated histone H3 (Cit-Histone 3) and myeloperoxidase (MPO), in myocardial tissue from the hypertensive animals relative to the Control group (**Figure 2D**). Consistently, ELISA assays of rat serum revealed significantly elevated Cit-Histone 3 (**Figure 2E**), MPO (**Figure 2F**), and neutrophil elastase (NE) (**Figure 2G**) levels in the hypertensive animals. Western blot analysis of myocardial tissue confirmed these findings, showing higher Cit-Histone 3 and MPO protein expression in the hypertensive animals compared with the Control group (**Figures 2H–J**).

At the cellular level, qPCR analysis of H9c2 cells demonstrated significantly increased mRNA expression of fibrotic markers, including a-SMA, fibronectin, collagen II, and MMP3, in both the Model and NETs-treated groups compared with the Control group (**Figures 2K–N**). Western blotting further corroborated these results, showing elevated protein levels of α-SMA, fibronectin, collagen II, and MMP3 in the Model and NETs groups relative to controls (**Figure 2O**). These findings collectively indicate that hypertensive myocardium is characterized by increased NET formation and upregulation of fibrotic markers, both *in vivo* and *in vitro*.

### Ferroptosis-related transcriptional changes and NF-κB pathway activation in the hypertensive myocardium

Accumulating evidence indicates that hypertensive stress is associated with myocardial ferroptosis and adverse cardiac remodeling. Reviews of cardiovascular ferroptosis consistently include hypertensive heart disease among conditions in which ferroptotic injury has been observed, and experimental models of pressure overload demonstrate increased ferroptotic death in cardiomyocytes with progression to fibrosis (38, 39). Angiotensin II–driven cardiac hypertrophy, a canonical hypertension model, likewise shows cardioprotective effects when ferroptosis is constrained, supporting a link between hypertensive stimuli and ferroptotic loss of myocardial cells (40). In parallel, inflammatory signaling appears to intersect with this process: activation of NF-κB has been reported to accompany and promote ferroptosis in cardiac contexts, and inhibition of this pathway attenuates ferroptosis-related cardiac injury in preclinical studies (30).

**Figure 3:**
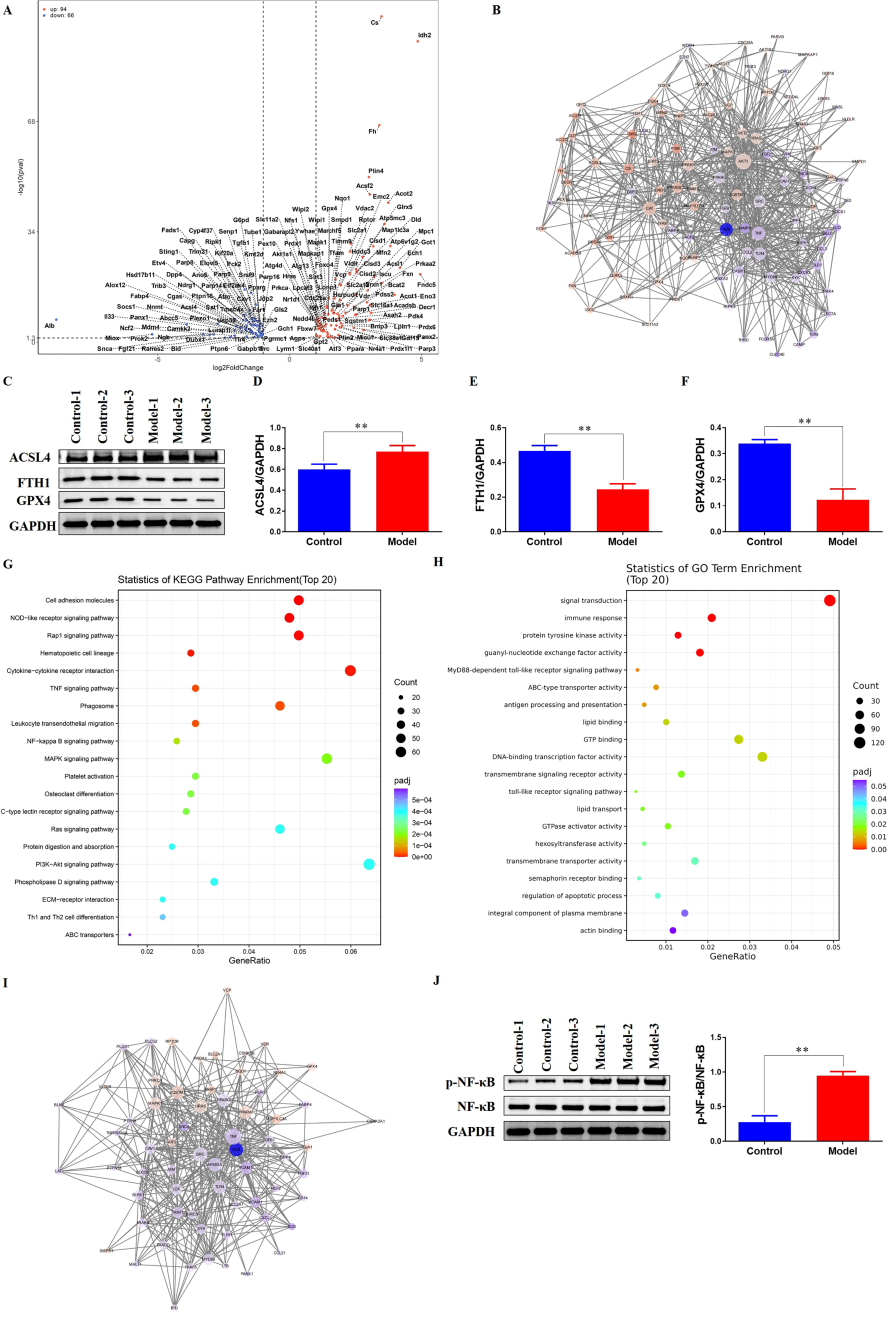
Coordinated ferroptosis and innate immune signaling in hypertensive myocardium. **(A)** Volcano plot of myocardial transcriptomic changes in the hypertensive animals versus Controls. **(B)** Protein-protein interaction (PPI) network analysis demonstrating reciprocal connectivity between ferroptosis-related and NETs-related gene modules. **(C)** Western blot analysis of ferroptosis markers. **(D)** Quantitative analysis of ACSL4 protein expression in myocardial tissue (**P < 0.01). **(E)** Quantitative analysis of FTH1 protein expression in myocardial tissue (**P < 0.01). **(F)** Quantitative analysis of GPX4 protein expression in myocardial tissue (**P < 0.01). **(G)** KEGG pathway enrichment analysis showing significant enrichment of the NF-κB signaling pathway among differentially expressed genes. **(H)** GO enrichment analysis indicating enrichment of the MyD88-dependent toll-like receptor signaling pathway and the toll-like receptor signaling pathway. **(I)** PPI network analysis integrating ferroptosis-related genes with NF-κB pathway genes, revealing interrelated networks. **(J)** Western blot analysis demonstrating higher activation of the NF-κB pathway in the Model myocardium compared to Controls (**P < 0.01).

Transcriptomic profiling clearly distinguished the hypertensive model group from the control group and revealed broad ferroptosis-related remodeling. A volcano plot highlighted substantial shifts in ferroptosis-associated transcripts, with 94 genes upregulated and 66 downregulated in the Model myocardium relative to Controls (**Figure 3A**).

To interrogate potential crosstalk with NETs, we performed a protein–protein interaction analysis that integrated NETs-related and ferroptosis-related gene sets. This analysis indicated reciprocal connectivity between the two modules (**Figure 3B**). Protein-level assessment in rat myocardium further supported ferroptosis involvement: Western blotting showed a marked increase of ACSL4 and reductions of FTH1 and GPX4 in the hypertensive animals compared with Controls (**Figures 3C–F**).

Pathway enrichment of differentially expressed genes corroborated inflammatory signaling engagement. KEGG analysis demonstrated enrichment of the NF-κB signaling pathway (**Figure 3G**), while GO analysis indicated enrichment of the MyD88-dependent toll-like receptor signaling pathway and the broader toll-like receptor signaling pathway (**Figure 3H**). Consistent with these findings, a PPI analysis integrating ferroptosis-related genes with NF-κB pathway genes revealed interrelated networks (**Figure 3I**).

Finally, Western blot analysis demonstrated higher activation of the NF-κB pathway in the Model myocardium than in Controls (**Figure 3J**). Collectively, these data delineate coordinated transcriptomic and proteomic alterations that couple ferroptosis signatures with NF-κB–centered innate immune signaling in hypertensive cardiac tissue.

### NETs promote a ferroptosis-prone phenotype and activate NF-κB signaling in H9c2 cells

**Figure 4:**
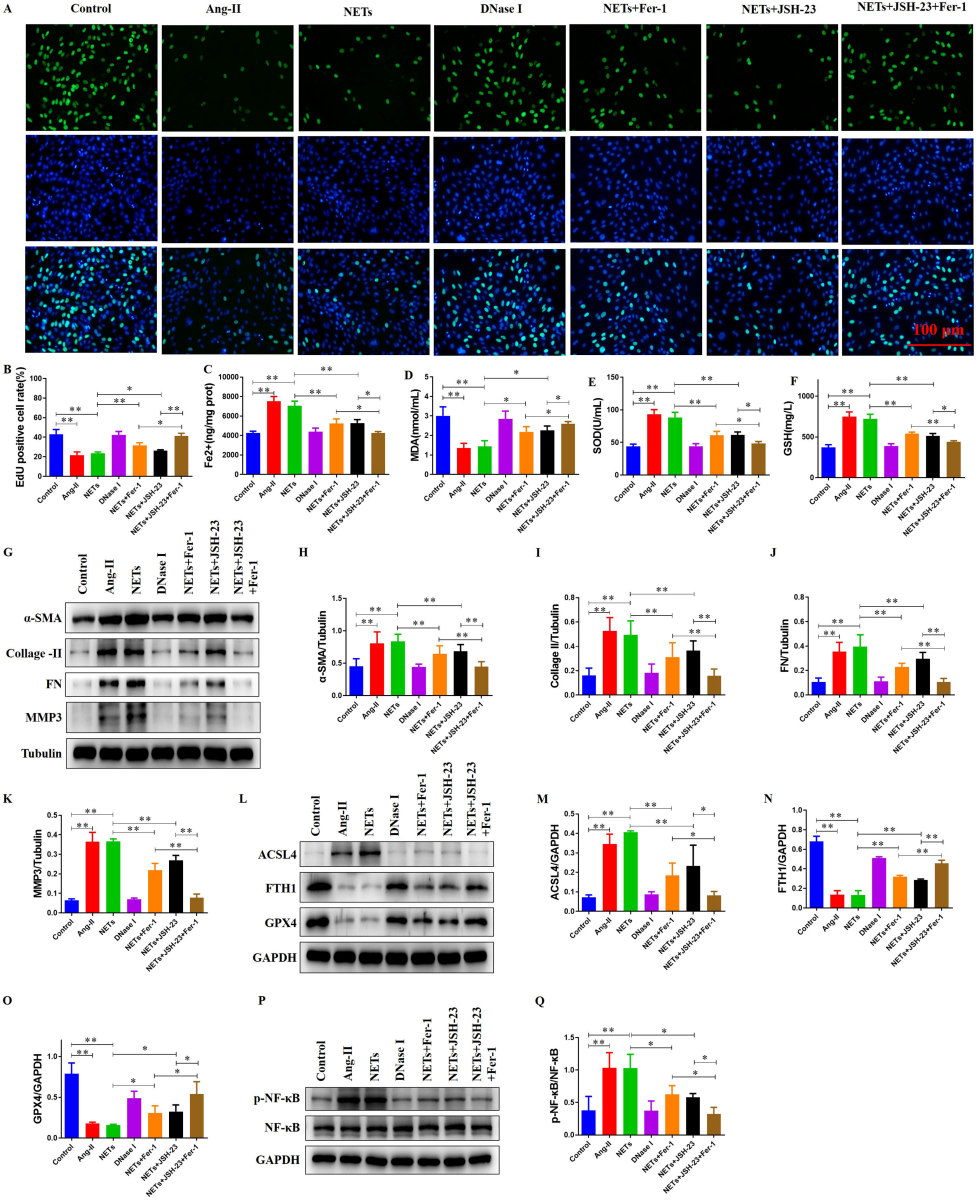
NETs promote a ferroptosis-prone phenotype, activate NF-κB signaling, and induce profibrotic protein expression in H9c2 cells. **(A)** Representative immunofluorescence images showing EdU proliferating nuclei and DAPI-stained total nuclei under indicated treatments. **(B)** Quantitative analysis of EdU^+^ cell ratio (*P < 0.05, **P < 0.01). **(C–F)** NETs induce ferroptotic responses: **(C)** Increased intracellular Fe²^+^ (*P < 0.05, **P < 0.01), **(D)** decreased MDA (*P < 0.05, **P < 0.01), **(E)** increased SOD activity (*P < 0.05, **P < 0.01), and **(F)** elevated GSH (*P < 0.05, **P < 0.01). Dysregulation is rescued by DNase I pretreatment, Fer-1, JSH-23, or combined inhibitors. **(G)** Representative Western blot analysis of fibrotic markers in H9c2 cells under indicated treatments. **(H)** Quantitative analysis of α-SMA protein expression in H9c2 cells (**P < 0.01). **(I)** Quantitative analysis of Collagen II protein expression in H9c2 cells (**P < 0.01). **(J)** Quantitative analysis of FN protein expression in H9c2 cells (**P < 0.01). **(K)** Quantitative analysis of MMP3 protein expression in H9c2 cells (**P < 0.01). **(L)** Western blot analysis of ferroptosis markers ACSL4, FTH1, and GPX4 in H9c2 cells under indicated conditions. **(M)** Quantitative analysis of ACSL4 protein expression in H9c2 cells (*P < 0.05, **P < 0.01). **(N)** Quantitative analysis of FTH1 protein expression in H9c2 cells (**P < 0.01). **(O)** Quantitative analysis of GPX4 protein expression in H9c2 cells (*P < 0.05, **P < 0.01). **(P)** Western blot analysis of NF-κB pathway activation in H9c2 cells under indicated conditions. **(Q)** Quantitative analysis of the p-NF-κB/NF-κB ratio densitometry derived from blots (*P < 0.05, **P < 0.01).

To determine whether NETs influence ferroptosis-related changes and cell proliferation in H9c2 cardiomyoblasts, we first assessed DNA synthesis by EdU staining (**Figure 4A**) and key redox/iron indices. Compared with the Control group, Ang II-treated group showed a significant decrease in EdU incorporation, and isolated NETs similarly suppressed proliferation; pre-treatment of NETs with DNase I restored proliferative activity toward control levels. Pharmacologic inhibition of ferroptosis with Ferrostatin-1 (Fer-1) or inhibition of NF-κB with JSH-23 each produced a significant recovery of proliferation in NETs-treated cells, and the combined JSH-23 + Fer-1 treatment yielded a further, significant increase relative to either single treatment (**Figure 4B**). Intracellular iron concentration was significantly elevated in the Ang II-treated group and NETs group versus Control, whereas DNase I-pretreated NETs normalized iron levels. Both Fer-1 and JSH-23 significantly reduced NETs-induced iron accumulation, and the combined treatment produced a larger reduction than either agent alone (**Figure 4C**). Measurements of oxidative status revealed that MDA content was reduced and SOD and GSH contents were increased in the Ang II-treated group and NETs group relative to Control; DNase I-treated NETs restored these indices toward control values. Treatment with Fer-1 or JSH-23 increased MDA and decreased SOD and GSH compared with NETs alone, with the combined regimen producing greater changes than either single agent (**Figures 4D–F**).

Consistent with the biochemical profile, protein analysis by Western blot showed that NETs exposure increased the pro-ferroptotic enzyme ACSL4 and decreased the iron-storage protein FTH1 and antioxidant enzyme GPX4, changes that mirrored those seen in the Ang II-treated group and NETs-treated group and were reversed by DNase I-pretreated NETs. Fer-1 or JSH-23 each attenuated NETs-induced upregulation of ACSL4 and partially restored FTH1 and GPX4 levels; the combined Fer-1 + JSH-23 treatment produced a further restoration compared with single treatments (**Figures 4G–K**). NETs also induced marked increases in fibrotic markers (α-SMA, Collagen II, Fibronectin and MMP3); these elevations were abolished by DNase I pretreatment and were significantly reduced by Fer-1 or JSH-23, with the strongest suppression observed with the combined treatment (**Figures 4L–O**). Finally, NETs markedly activated the NF-κB signaling pathway in H9c2 cells (**Figure 4P**); DNase I-pretreated NETs returned NF-κB activation to control levels, and both Fer-1 and JSH-23 suppressed NETs-induced NF-κB activation, with the combined inhibition producing the greatest decrease (**Figure 4Q**). Collectively, these findings indicate that NETs promote a ferroptosis-prone molecular state, oxidative imbalance and profibrotic protein expression in H9c2 cells and that NET degradation or inhibition of ferroptosis and NF-κB signaling mitigates these effects.

## Discussion

This study provides compelling evidence that NETs play a pivotal role in the pathogenesis of myocardial fibrosis in hypertensive animals by orchestrating ferroptosis and activating NF-κB signaling. Through a combination of *in vivo* and *in vitro* analyses, we delineated a mechanistic axis wherein NETs promote cardiac fibrosis via induction of oxidative stress, ferroptotic cell death, and pro-fibrotic gene expression—processes that are significantly mitigated by pharmacological inhibition of either ferroptosis or NF-κB.

The elevation in blood pressure and reduction in left ventricular function observed in hypertensive animals are consistent with the well-documented cardiovascular phenotype associated with spontaneously hypertensive rats (41, 42). Histological examination confirmed extensive myocardial remodeling characterized by collagen accumulation and fibroblast activation, corroborated by the upregulation of canonical fibrosis markers such as α-SMA, fibronectin, collagen type II, and MMP3. These findings align with prior reports emphasizing the centrality of myocardial fibrosis in hypertensive heart disease and highlight the clinical relevance of the spontaneously hypertensive model in recapitulating human pathology (43–45). Additionally, our echocardiographic data revealed significant impairment in LVEF and FS in SHRs, indicative of systolic dysfunction. This aligns with the clinical trajectory of human HHD, where chronic pressure overload initially leads to diastolic dysfunction but often progresses to encompass systolic impairment, especially in advanced stages or under sustained stress. The observed systolic dysfunction in our model likely results from a combination of factors: the direct loss of cardiomyocytes via NETs-promoted ferroptosis, which reduces contractile unit mass; the interstitial fibrosis disrupting myocardial syncytium architecture and impairing force transmission; and the potential functional compromise of surviving cardiomyocytes due to inflammatory and oxidative stress.

A key finding of this study is the enhanced NET formation in hypertensive animals, evidenced by increased myocardial and serum levels of Cit-H3 and MPO. NETs have been increasingly recognized as pathogenic entities beyond their antimicrobial function, with implications in atherosclerosis, autoimmune diseases, and cardiac injury (46–48). Our transcriptomic analysis revealed differential expression of NET-related genes in hypertensive myocardium, supporting their active role in disease progression. Moreover, the application of NETs to H9c2 cardiomyoblasts recapitulated fibrotic phenotypes observed *in vivo*, indicating that NETs are not merely a byproduct of tissue injury but active upstream drivers of fibrotic remodeling. From a translational perspective regarding systolic function, our mechanistic findings suggest that NETs could contribute to human hypertensive systolic dysfunction through similar pathways. In patients with HHD, elevated circulating NETs markers might correlate not only with fibrosis burden but also with the degree of systolic impairment. The NETs/NF-κB/ferroptosis axis identified here provides a plausible molecular link between sterile inflammation, cardiomyocyte loss, and the deterioration of contractile performance. Pharmacological strategies targeting this axis could therefore have dual benefits: attenuating fibrotic remodeling and potentially preserving or rescuing systolic function by preventing cardiomyocyte ferroptotic death and mitigating associated inflammation.

However, it is important to acknowledge the limitations of the H9c2 cell line in fully representing the complexity of adult cardiomyocyte biology and the cardiac microenvironment. H9c2 cells, while widely used as a model for cardiomyocytes, are derived from embryonic rat heart tissue and exhibit characteristics of undifferentiated cardiomyoblasts rather than mature cardiomyocytes. They lack the mature electrophysiological properties, such as organized sarcomeres and functional gap junctions, that are essential for proper cardiac function. Moreover, the H9c2 cell line does not recapitulate the intricate cellular crosstalk within the myocardial microenvironment, which includes interactions between cardiomyocytes, cardiac fibroblasts, endothelial cells, and immune cells. These limitations may affect the interpretation of our findings regarding NETs-induced ferroptosis and fibrotic signaling. For instance, the observed effects on ferroptosis markers and NF-κB activation in H9c2 cells might not fully reflect the responses in mature cardiomyocytes within the intact myocardium. To address these limitations, future studies should incorporate more physiologically relevant models, such as primary cardiomyocytes derived from adult rat hearts or human induced pluripotent stem cell-derived cardiomyocytes (hiPSC-CMs), as well as co-culture systems that better mimic the cardiac microenvironment. Additionally, validation of our findings in more complex *in vivo* models, such as conditional knockout mice targeting NETs formation or ferroptosis pathways in cardiomyocytes, would strengthen the translational relevance of our observations.

Mechanistically, our study demonstrates that NETs induce ferroptosis in cardiomyocytes, characterized by increased intracellular iron levels, upregulation of ACSL4, and concomitant downregulation of the ferroptosis-suppressive proteins FTH1 and GPX4. This iron-dependent form of regulated cell death has emerged as a novel modality contributing to cardiac injury, yet its role in NET-mediated fibrosis had not been explicitly demonstrated prior to this investigation. Importantly, DNase I pre-digestion of NETs abrogated ferroptotic responses, implicating NET-associated DNA structures as essential effectors in this pathway. This finding strengthens the notion that extracellular chromatin scaffolds and histone-associated proteins are not inert byproducts, but bioactive mediators capable of reprogramming cardiomyocyte survival and redox balance.

However, the precise molecular identity of the NET components initiating ferroptosis remains to be fully elucidated. Our data show that DNase I digestion—which disrupts the NET backbone—abolishes ferroptotic signaling, suggesting that the structural integrity or DNA-histone complex is critical. Beyond the scaffold, specific protein components such as citrullinated histones or myeloperoxidase may directly inflict oxidative damage or activate pattern recognition receptors on cardiomyocytes, leading to iron overload and lipid peroxidation. Future studies using recombinant NET constituents or selective blockade of their cognate receptors will be necessary to dissect which components are sufficient and necessary for ferroptosis induction, and through which downstream sensors they act.

The NF-κB signaling axis emerged as a critical mediator linking NETs and ferroptosis to fibrosis (49). KEGG enrichment of differentially expressed genes and Western blotting of myocardial tissues confirmed the activation of NF-κB in hypertensive animals. This pathway has been well-established in orchestrating inflammatory and fibrotic transcriptional programs in cardiovascular disease (50, 51). Our data show that both pharmacological inhibition of NF-κB (via JSH-23) and suppression of ferroptosis (via Fer-1) individually attenuated NETs-induced fibrotic and ferroptotic markers in H9c2 cells. Notably, their combined administration exerted synergistic effects, indicating potential crosstalk between these signaling cascades. This synergism suggests that ferroptosis and NF-κB activation form a self-reinforcing feed-forward loop, wherein iron-driven lipid peroxidation amplifies NF-κB activity, which in turn promotes inflammatory gene expression and further sensitizes cardiomyocytes to ferroptosis.

We acknowledge that the molecular hierarchy between NET recognition, NF-κB activation, and ferroptosis execution warrants further dissection. For instance, it remains unclear whether NETs activate NF-κB directly via TLR9 or other DNA-sensing pathways, which then transcriptionally regulate ferroptosis-related genes, or whether ferroptotic lipid peroxides themselves activate NF-κB as a secondary stress response. Detailed time-course experiments and pathway-specific genetic manipulations could help resolve this temporal and mechanistic sequence.

From a translational perspective, these findings have important implications. First, NETs may serve as both biomarkers and therapeutic targets for hypertensive myocardial remodeling. Circulating Cit-H3 and MPO could provide accessible indices of disease activity, while interventions aimed at NET degradation (e.g., DNase I, PAD4 inhibitors) may attenuate cardiac fibrosis. Second, the demonstration of functional convergence between ferroptosis and NF-κB provides a rationale for combination therapies—such as antioxidants, ferroptosis inhibitors, and anti-inflammatory agents—that could act synergistically to blunt fibrotic progression in HHD. Critically, such therapeutic strategies might also address systolic dysfunction. By curbing cardiomyocyte loss via ferroptosis inhibition and reducing the inflammatory milieu via NF-κB suppression, these interventions could help preserve the contractile myocardium. The partial restoration of cell proliferation in our *in vitro* model upon combined inhibition hints at a potential for cellular repair or reduced attrition. While reversal of established fibrosis and systolic impairment is challenging, early or concurrent targeting of the NETs/NF-κB/ferroptosis axis could prevent the transition from compensated hypertrophy to systolic failure. Future studies should directly evaluate the impact of axis-specific inhibitors on echocardiographic parameters of systolic function in longitudinal hypertensive models. Third, our results expand the conceptual framework of hypertensive cardiac pathology from hemodynamic overload alone to include immune–cell death crosstalk as a fundamental driver of maladaptive remodeling.

Nonetheless, several limitations must be acknowledged. First, while the H9c2 cell line provides a robust model for cardiomyoblast behavior, it does not fully replicate the complex interactions within the myocardial microenvironment, including immune cell crosstalk and hemodynamic stress. Second, although our data support causality between NETs and ferroptosis, future studies employing genetic models would further substantiate these relationships. In particular, a more granular dissection of the NET components and their cognate signaling receptors in cardiomyocytes is needed to establish a definitive molecular link. Finally, the translational relevance of these findings to human hypertensive heart disease warrants validation in clinical specimens or large-animal models. Specifically concerning systolic function, our study does not establish a direct causal link between the NETs/NF-κB/ferroptosis axis and the measured systolic dysfunction in SHRs, as we did not perform functional rescue experiments *in vivo* with axis-specific inhibitors. The observed systolic impairment is likely multifactorial. Furthermore, the SHR model at the studied timepoint primarily exhibits preserved ejection fraction with diastolic dysfunction, and the observed reduction in LVEF/FS, while significant, may represent an early systolic compromise. The relevance of our axis to the more profound systolic dysfunction seen in human HFrEF needs dedicated investigation. Moreover, longitudinal studies are required to determine whether inhibition of the NETs/NF-κB/ferroptosis axis not only prevents fibrosis but also reverses established fibrotic remodeling and improves systolic performance.

In conclusion, this study elucidates a novel mechanistic link between NETs, NF-κB signaling, and ferroptosis in the pathogenesis of myocardial fibrosis under hypertensive conditions. Our findings, coupled with the observed systolic dysfunction in the model, suggest that this pathogenic axis may contribute to the broader spectrum of cardiac functional decline in HHD, encompassing both diastolic and systolic components. By disrupting this pathogenic axis, it may be possible to attenuate fibrotic remodeling and potentially mitigate the progression of systolic dysfunction, thereby improve cardiac outcomes in patients with hypertensive heart disease. Our findings therefore highlight the NETs/NF-κB/ferroptosis axis as both a mechanistic insight and a translationally relevant therapeutic target, bridging basic discovery and potential clinical intervention.

## Data Availability

The original contributions presented in the study are publicly available. This data can be found here: http://db.cngb.org/cnsa/project/CNP0007673_629f2b6f/reviewlink/.
